# A Protocol for a Novel Human *Ex Vivo* Model of Aneurysm

**DOI:** 10.1016/j.xpro.2020.100108

**Published:** 2020-09-09

**Authors:** Rosaria Bianco, Karina Di Gregoli, Massimo Caputo, Sarah J. George, Jason L. Johnson

**Affiliations:** 1 Laboratory of Cardiovascular Pathology, Department of Translational Health Sciences, Bristol Medical School, Faculty of Health Sciences, University of Bristol, Bristol, UK

## Abstract

Aortic aneurysm rupture is a significant cause of premature mortality worldwide. Although animal models exist, some frequently experience aortic rupture and sudden death. An alternative approach is therefore required that would use human material to aid translation. Accordingly, we present an optimized and validated protocol to isolate human umbilical cord arteries and their subsequent deployment within a bioreactor. Consequently, this reproducible *ex vivo* human model of aneurysm can be used for pathogenesis studies and accompanying assessment of potential novel therapeutics.

## Highlights

• The use of human umbilical cord artery negates the need for animal experimentation
• The use of human umbilical cord artery provides translational potential
• Angiotensin II and pulsatile flow induce vessel dilation and early aneurysm formation
• This model displays histological characteristics with those observed in human aneurysms

## Graphical Abstract

**Figure:**
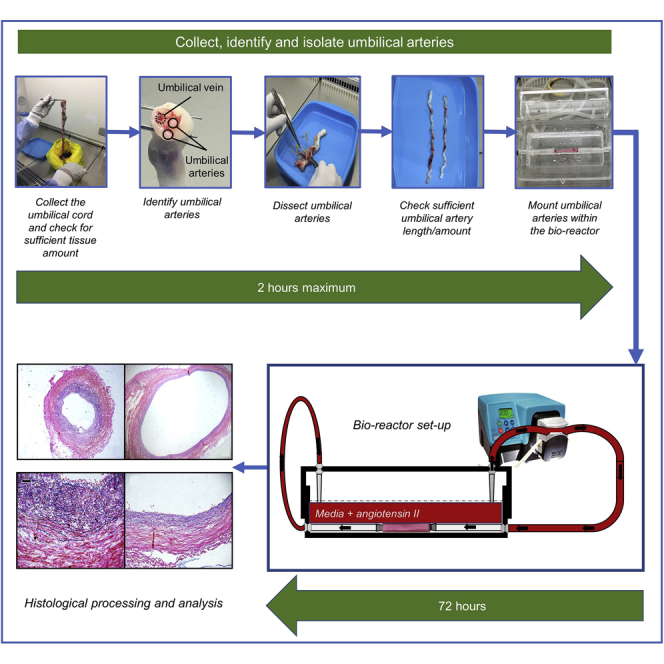

## Before You Begin

The establishment of the *ex vivo* model of aneurysm formation requires the bioreactor to be set up, following these major steps:

### Experimental Design Consideration

**Timing: 2 h**

1. Arrange umbilical cord delivery with the research nurse at the Maternity Hospital.
2. Arrange umbilical cord delivery with other research groups within the department.
3. Plan the experimental design in order to know how many chambers, lengths of tubing, and number of canulae are required.
4. Plan the conditions of scheduled treatments and interventions to be used and therefore the number of chambers that need to be set up.
5. Calculate the shear stress to be used. In abdominal aortic aneurysm (AAA) patients, the wall shear stress is between 3.6 to 9.2 dyn/cm^2^. Accordingly, we use a wall shear stress of 6.5 dyn/cm^2^ in a pulsatile laminar flow direction within the *ex vivo* model to ensure similar haemodynamic conditions to the abdominal segment of the human aorta.

### Preparation of Bioreactor Components

**Timing: 2 h**

6. Tubing, autoclave, and medium
a. Cut long silicon tubes into the correct length and connect to a stronger silicon tube. This additional strength is required to be used within the peristaltic pump. Connect all tubes together with cannula at both ends and then autoclave.
b. Cut short silicon tubes, connect to cannula at both ends and then autoclave.
c. Autoclave water, forceps, and sylgard dishes.
d. Obtain Dulbecco’s Modified Eagle's Medium (DMEM) and supplement with gentamicin, penicillin, streptomycin, and L-glutamine.
e. Prepare a fresh formaldehyde.

### Assembly of Bioreactor

**Timing: 30 min**

**Figure 1:**
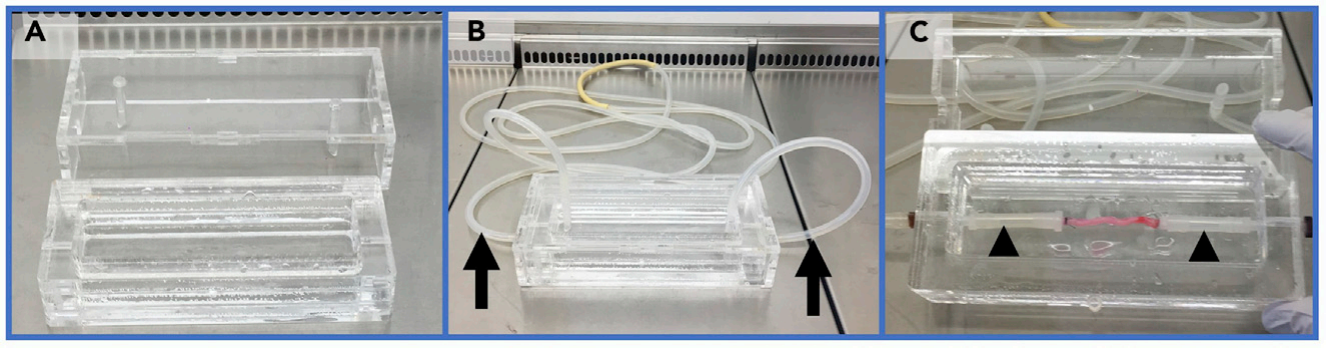
Bioreactor Set Up (A) Bioreactor chamber under hood. (B) Long silicon tube connected at both ends to the bioreactor chamber (see arrows). (C) Two short silicon tubes connected to the inner edges of the bioreactor chamber (see arrowheads).
b. Connect a long silicon tube at both ends to the bioreactor chamber (Figure 1B).
c. Connect two short silicon tubes to the inner edges of the bioreactor chamber (Figure 1C).

7. Bioreactor
a. Place bioreactor chambers for at least 2 h in a Milton sterilizing solution, briefly wash in sterile water and then place under a tissue culture hood to dry (Figure 1A).

## Key Resources Table

**Table:** 

REAGENT or RESOURCE	SOURCE	IDENTIFIER
**Biological Samples**		

Umbilical cord arteries from human umbilical cord	St. Michael’s Maternity Hospital	N/A

**Chemicals, Peptides, and Recombinant Proteins**		

Angiotensin II	Enzo Life Sciences	Cat#ALX-151-039
Dulbecco’s modified Eagle’s medium (DMEM)	Sigma	Cat#D5546
Fetal bovine serum (FBS)	ThermoFisher	Cat#10500-064
Gentamicin	Sigma	Cat#G1397
Penicillin and streptomycin	Sigma	Cat#P4333
L-glutamine	Sigma	Cat#G7513
PBS	Sigma	Cat#D8537
Chloroform	Sigma	Cat#132950
Clearene (xylene substitute)	Leica	Cat#3803600
100% alcohol	Leica	Cat#3803686
Potassium permanganate	VWR	Cat#26904.293
Oxalic acid	VWR	Cat#0326-500G
Millers elastin stain	VWR	Cat#351154S
Van Gieson stain	ThermoFisher	Cat#LAMB-400-D
Mayer’s hemotoxylin	VWR	Cat#1004710S
Scotts tap water	Sigma	Cat#S5134
Eosin Y	VWR	Cat#341972Q
Picrosirius red solution	Abcam	Cat#ab246832
Hydrochloric acid	Sigma	Cat#320331
Citric acid	Sigma	Cat#251275
DPX mounting medium	Sigma	Cat#06522
BLOXALL endogenous blocking solution	Vector Labs	Cat#SP-6000
ImmEdge Pen	Vector Labs	Cat#H-400
Horse serum	Vector Labs	Cat#S-2000
Streptavidin, DyLight-488 conjugated	Vector Labs	Cat#SA-5488
Vectashield antifade mounting medium with DAPI	Vector Labs	Cat#H-1500

**Software and Algorithms**		

Image Pro Plus v7.0	Media Cybernetics	http://www.mediacy.com/imageproplus
Image J	NIH	https://imagej.nih.gov/ij/

**Other**		

Suture, Mersilk 5-0 black	Ethicon	Cat#W500H
Cannula, Male Luer Fitting for 1/16 inch ID Tubing	World Precision Instruments	Cat#13160-100
Wacker silicon tubing, ID 3.18MM, OD 6.35MM	VWR	Cat#228-1094
Watson-Marlow Sci-Q 300 Series Peristaltic Pump	ThermoFisher	Cat#14-284-11
Minisart syringe filter	VWR	Cat#516-0907
Humidified chamber	Sigma	Cat#2670138

**Antibodies**		

Monoclonal anti-alpha-smooth muscle actin antibody	Sigma	Cat#A2547-.2ML
Monoclonal anti-ER-TR7 antibody	Bio-Techne	Cat#NB100-64932
Horse anti-mouse biotinylated IgG antibody	Vector Labs	Cat#BP-2000
Goat anti-rat biotinylated IgG antibody	Vector Labs	Cat#BP-9400
Mouse IgG	Vector Labs	Cat#I-2000
Rat IgG	Vector Labs	Cat#I-4000

## Materials and Equipment

**CRITICAL:** All preparatory procedures need to be performed within a Class II biological hood with standard aseptic technique.

### Serum Free Dulbecco’s Modified Eagle's Medium (SFM)

Serum free Dulbecco’s Modified Eagle's Medium (DMEM) is supplemented as detailed below. All supplements are added using a Minisart syringe filter to prevent fungal and bacterial infections, mixed well and finally stored at 4°C.

**Table:** Media composition for Serum Free DMEM

Reagent	Final Concentration	Amount
Gentamicin	400 μL/500 mL	100 μL
Penicillin and Streptomycin	100 μL/mL and100 μL/mL	5 mL
L-glutamine	2 mM	5 mL
DMEM	n/a	500 mL

### 10% FBS/DMEM Culture Media

Serum free DMEM is supplemented with 50 mL fetal bovine serum (FBS) gold to obtain 10% FBS/DMEM. FBS is the most common growth supplement used for the *in vitro* and *ex vivo* cell culture. FBS has a high content of embryonic growth and therefore is always used for all our *in vitro and ex vivo* studies.

All the components of 10% FBS/DMEM are listed below. FBS is added using a Minisart syringe filter to prevent fungal and bacterial infections, mixed well and finally stored at 4°C.

**Table:** Media composition for 10% FBS/DMEM

Reagent	Final Concentration	Amount
Fetal bovine Serum	10%	50 mL
Gentamicin	400 μL/500 mL	100 μL
Penicillin and Streptomycin	100 μL/mL and 100 μL/mL	5 mL
L-glutamine	2 mM	5 mL
DMEM	n/a	450 mL

### Bioreactor Components

A Watson-Marlow Sci-Q 300 Series Peristaltic Pump from Thermo Fisher is used to generate a wall shear stress of 6.5 dyn/cm^2^ in a laminar flow direction to induce the *ex vivo* aneurysmal model. A Wacker silicon tubing, ID 3.18MM, OD 6.35MM from VWR is used to connect either ends of the bioreactor chamber. A Cannula, Male Luer Fitting for 1/16" ID Tubing from World Precision Instrument is connected to the silicon tubing and then placed inside a supporting bioreactor chamber. A Suture, Mersilk 5-0 black from Ethicon is used to tie the artery onto the cannula.

## Step-By-Step Method Details

**CRITICAL:** Human umbilical cord samples must be obtained in compliance with institutional review board regulations and ethical guidelines. All procedures used for handling human tissues should assume potential contamination of tissue with human pathogens (HBV, HCV, HIV, C. difficile, etc.).

### Dissection of Arteries from Human Umbilical Cord

**Timing: 2 h**

**Figure 2:**
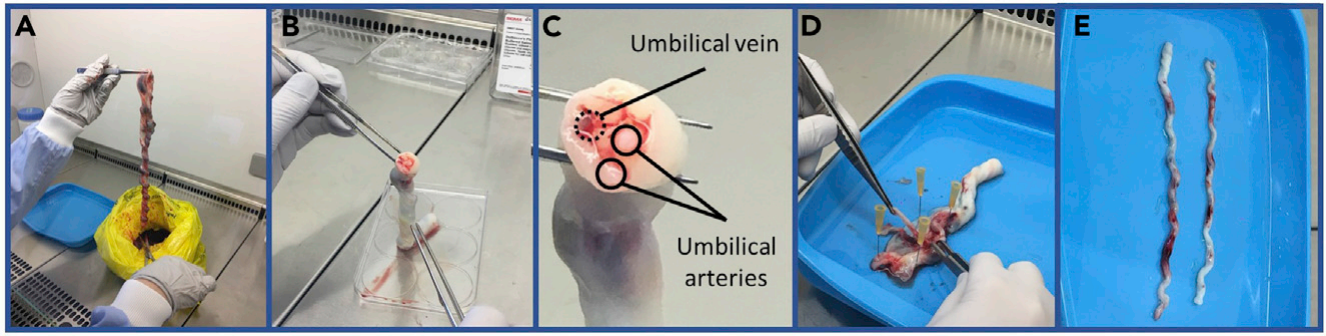
Method Detail (A) Human umbilical cords were obtained from St Michaels’ Maternity Hospital. (B) Arteries and vein were identified in umbilical cord cross-section. (C) The cord is partially pinned down to permit further dissection. (D) Arteries should be used immediately after dissection.

This step details how to dissect the arteries from human umbilical cord (as shown in Figure 2).

1. Obtain human umbilical cords, covered under existing ethical approval.
a. Store umbilical cords in serum free media at 4°C immediately after being obtained.
b. Dissect umbilical cord samples, limiting the time between harvest and usage within the bioreactor setup to 90 ± 30 min.
2. Human umbilical cords contain two arteries and one vein surrounded by Wharton jelly.
a. Under sterile conditions, place a length of umbilical cord within a sylgard petri dish.
b. Identify the two arteries and one vein by observing a cross-section of the umbilical cord.
3. Partially open the cord and pin down on the dish (a flat surface is required to work on).
4. Micro-dissect the arteries under phosphate-buffered saline (PBS, pH 7.4) containing penicillin 100 U/mL and streptomycin 100 mg/mL at 4°C and flush with PBS to remove any possible blood clots before transferring into serum free DMEM at 4°C
5. Use the excised arteries immediately after the dissection to preserve vessel integrity and maintain experimental consistency.

### *Ex Vivo* Model of Aneurysm

**Timing: 3 h**

This step details how to set up the bioreactor system for the *ex vivo* aneurysm model

6. Sterilize tissue culture hoods with a Milton sterilizing solution
7. Place bioreactor chambers in Milton sterilizing solution for at least 2 h, then briefly wash in sterile water and finally place under a tissue culture hood to dry.
8. Cut the arteries (stored in serum free DMEM at 4°C) into three equal lengths of 5 cm to be used for baseline, untreated, and treated conditions, respectively.
9. Connect the short silicon tubes at both ends of the bioreactor chamber.
10. Mount the arteries between two small cannulae, using sutures to double tie the artery onto each cannula.
11. Connect each cannula to the short silicon tube and then place inside a supporting bioreactor chamber.
12. Fill the long silicon tube with 10% FBS/DMEM and pass the tube through a peristaltic pump to close the system and permit the induction of laminar flow within an incubator.
13. Subject each chamber to identical pulsatile flow conditions with a flow rate of 6.5 dyn/cm^2^ to closely mimic abdominal aortic *in vivo* conditions, at 37°C, 5% CO_2_ for 72 h.
14. As a control, use untreated chambers with 80 mL of 10% FBS/DMEM. For Ang II-treated arteries, supplement 80 mL of 10% FBS/DMEM with 5 μM Angiotensin II. For intervention-treated chambers, add 80 mL of 10% FBS/DMEM with the corresponding intervention.
15. After 72 h of flow within the bioreactor chambers, carefully remove the arteries.
16. Remove and dispose of the two edges of the artery (not subjected to a laminar flow) where they were sutured to the cannula. Cut the artery cut into two segments, to be named as proximal and distal.
17. Fix the proximal segment in 10% formalin and prepare for histology and store the distal segment in a −80°C freezer for downstream genomic/proteomic analysis.

### Histological Processing: Elastin van Gieson Staining

**Timing: 3 h 30 min**

18. The proximal portion of the artery (approx. 2.5 cm in length) should be cut into five 5 mm rings for subsequent histological processing and wax embedding.
19. From the formalin-fixed wax-embedded arterial rings, cut 3 μm sections collected upon adhesive glass microscope slides (two sections per slide).
20. To enable assessment of arterial elastin content, perform Elastin van Gieson (EVG) histochemical staining using an automated stainer (such as a Shandon Varistain 24-4 Automatic Slide Stainer) or manual protocol (protocol details are shown in Table 1).

**Table 1:** Staining Protocol for EVG

Solution	Incubation Time
Xylene substitute (such as Clearene)	3 × 5 min
100% (v/v) alcohol (or industrial methylated spirits)	3 × 5 min
Tap water	5 min
0.5% (w/v) potassium permanganate	10 min
Distilled water	3 min
1% (w/v) oxalic acid	5 min
Distilled water	3 min
70% (v/v) alcohol (or industrial methylated spirits)	2 min
50% (v/v) Millers elastin stain (VWR, Cat#351154S)	60 min
70% (v/v) alcohol (or industrial methylated spirits)	2 min
Running tap water	3 min
Van Gieson stain (ThermoFisher, LAMB-400-D)	30 s
100% (v/v) alcohol (or industrial methylated spirits)	3 × 5 min
Xylene substitute (such as Clearene)	3 × 5 min
21. After EVG staining, coverslip sections using a relevant mounting medium (such as DPX) and visualize the sections using a bright field microscope and acquire images under ×20 magnification.
22. Determine the relative amount of elastin (which appears as black under a light microscope) using a computerized image analysis program (such as Image Pro Plus or ImageJ) and express as an average percentage of the arterial cross-sectional area.

### Histological Processing: Hematoxylin and Eosin Stain

**Timing: 1 h 30 min**

23. From the formalin-fixed wax-embedded arterial rings, cut 3 μm sections collected upon adhesive glass microscope slides (two sections per slide).
24. To enable assessment of arterial cellular content and general morphology, perform haematoxylin and eosin (H&E) histochemical staining using an automated stainer (such as a Shandon Varistain 24-4 Automatic Slide Stainer) or manual protocol (protocol details are shown in Table 2).

**Table 2:** Staining Protocol for H&E

Solution	Incubation Time
Xylene substitute (such as Clearene)	3 × 5 min
100% (v/v) alcohol (or industrial methylated spirits)	3 × 5 min
Tap water	5 min
Mayer’s Haematoxylin	3 min
Distilled water	3 min
Scott’s Tap water	1 min
Running tap water	3 min
1% Eosin	1 min
Running tap water	3 min
100% (v/v) alcohol (or industrial methylated spirits)	3 × 5 min
Xylene substitute (such as Clearene)	3 × 5 min
25. After H&E staining, coverslip sections using a relevant mounting medium (such as DPX) and visualize the sections using a bright field microscope and acquire images under ×20 magnification.
26. Count the number of nuclei and express as an average percentage of the arterial cross-sectional area.

### Histological Processing: Picrosirius Red Staining

**Timing: 4 h**

27. From the formalin-fixed wax-embedded arterial rings, cut 3 μm sections collected upon adhesive glass microscope slides (two sections per slide).
28. To enable assessment of arterial collagen content, perform picrosirius red (0.1% (w/v) Sirius Red F3B saturated aqueous picric acid, pH 1.8–2.2) histochemical staining (protocol details are shown in Table 3).

**Table 3:** Staining Protocol for Picrosirius Red

Solution	Incubation Time
Xylene substitute (such as Clearene)	3 × 5 min
100% (v/v) alcohol (or industrial methylated spirits)	3 × 5 min
Distilled water	3 × 2 min
Picrosirius red solution 0.1% (w/v)	90 min
0.01 N hydrochloric acid	2 × 15 s
Distilled water	2 × 2 min
Oven dry at 37°C	80 min
100% (v/v) alcohol (or industrial methylated spirits)	1 × 5 min
Xylene substitute (such as Clearene)	3 × 5 min
29. After picrosirius red staining, coverslip sections using a relevant mounting medium (such as DPX) and visualize the sections using a bright field polarizing microscope and acquire images under ×20 magnification.
30. Determine the relative amount of collagen (which appears as red under a light microscope) using a computerized image analysis program and express as an average percentage of the arterial cross-sectional area. Qualitative analysis of fiber thickness/age is assessed by delineating green and red fibers indicated under polarized light, as fiber color variation transits from green to red proportional to the increase of fiber thickness/age. The relative amount of each fiber color is expressed as a percentage of the total amount of collagen in the area of interest.

**CRITICAL:** Ensure slides are dry before placing in 100% alcohol toward the end of the staining, as any residual water will cause the Picrosirius Red stain to leach from the section.

### Histological Processing: Immunohistochemistry for Alpha-Smooth Muscle Actin and ER-TR7

**Timing: 1 day**

31. From the formalin-fixed wax-embedded arterial rings, cut 3 μm sections collected upon adhesive glass microscope slides (two sections per slide).
32. To enable assessment of vascular smooth muscle cell phenotypic modulation and density, and assess fibroblast presence, perform immunohistochemistry for alpha-smooth muscle actin (αSMactin) and ER-TR7 expression, respectively.

Dewax sections by placing in three changes of Clearene for the duration of 5 min in each.

33. Rehydrate sections by placing in three changes of graduated alcohol (100%, 90% and 70%) for the duration of 5 min in each.
34. Rinse in distilled H_2_O.
35. Place slides in a black trough with 10 mM citrate acid pH 6.0 (2.1 g/L distilled H_2_O)
36. 6 min full power in microwave
37. Top up with distilled water
38. 6 min full power in microwave
39. Top up with distilled water
40. Leave to cool for 30 min
41. Rinse in phosphate-buffered saline (PBS) for 3 × 2 min.
42. Dry back of slide with tissue paper and encircle sections with wax pen (ImmEdge Pen; Vector Labs; cat#H-400), place within a humidified chamber and then inhibit endogenous peroxidase activity with Bloxall endogenous blocking solution for 10 min at 18°C–22°C.
43. Rinse in PBS for 3 × 2 min, then pipette on 50 μL of 10% horse serum.
44. Incubate at 18°C–22°C for 30 min.
45. Tap off solution, dry back of slides and pipette on 50 μL of either mouse anti-human alpha-smooth muscle actin (1/200) or rat anti-human ER-TR7 (1/250), diluted in PBS. Use same species IgG diluted to same concentration as primary to act as a negative control.
46. Incubate for 16–18 h at 4°C.
47. Wash sections in PBS for 3 × 2 min.
48. Dry back of slides with tissue and pipette onto sections 50 μL of either horse anti-mouse biotinylated ready-to-use antibody (Vector Labs; cat#BP-2000) for alpha-smooth muscle actin detection, or goat anti-rat biotinylated ready-to-use antibody (Vector Labs; cat#BP-9400) for ER-TR7 detection, diluted 1/200 in PBS.
49. Incubate at 18°C–22°C for 30 min.
50. Wash sections in PBS for 3 × 2 min.
51. Dry back of slides with tissue and pipette onto sections 50 μL DyLight-488-conjugated Streptavidin (10 μg/mL; Vector Labs; cat#SA-5488).
52. Incubate at 18°C–22°C for 1 h.
53. Wash sections in PBS for 3 × 2 min.
54. Mount slides with appropriately sized coverslip and Vectashield antifade mounting medium with DAPI (Vector Labs; cat#H-1500) to enable the labeling of nuclei.

## Expected Outcomes

**Figure 3:**
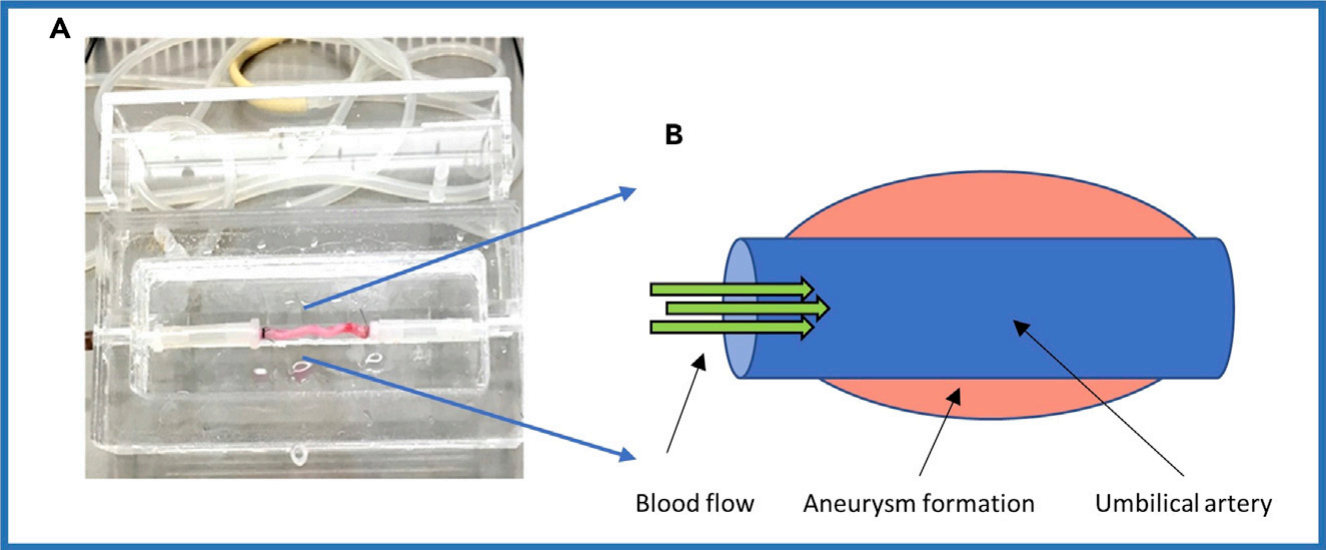
Summary of Our *Ex Vivo* Aneurysm Model (A) Arteries isolated from human umbilical cords were mounted between two small cannulae within a bioreactor chamber. (B) The principle of the *ex vivo* model is delivery of laminar flow conditions (green arrow) alongside angiotensin II within the artery to generate concentric dilatation (pink).

Following the above method, we have developed (and characterized) a reproducible *ex vivo* model of aneurysm formation with the use of human umbilical cord arteries stimulated with angiotensin II within a bioreactor system (summarized in Figure 3). Angiotensin II (Ang II) infusion is the most commonly used approach to induce aneurysm formation in animal models, especially mice, due to its ability to promote aneurysm formation with the most comparable histological features to those observed within human aneurysms (Sénémaud et al, 2017). Accordingly, angiotensin II infusion was therefore selected for use in our *ex vivo* model. The concentration of Ang II was established on the basis of previously published Ang II-infusion mouse model studies (Daugherty et al., 2000, Di Gregoli et al., 2017). Importantly, the dose used in our model was selected according to the range detected in human aneurysmal disease. The circulating Ang II level within healthy human subjects is between 4 and 15 pg/mL whereas AAA patients display plasma Ang II levels of 20–85 pg/mL. Within the *ex vivo* bioreactor model a concentration of 65 pg/mL was used, which is within the range detected in AAA patients.

Morphological and compositional changes typical of aneurysm formation have been widely characterized (Davies, 1998, Michel et al., 2011, Nordon et al., 2011), therefore a good model for aneurysm studies should display such characteristics, which includes vessel dilatation, medial thinning, elastin degradation, and loss of vascular smooth muscle cells.

**Figure 4:**
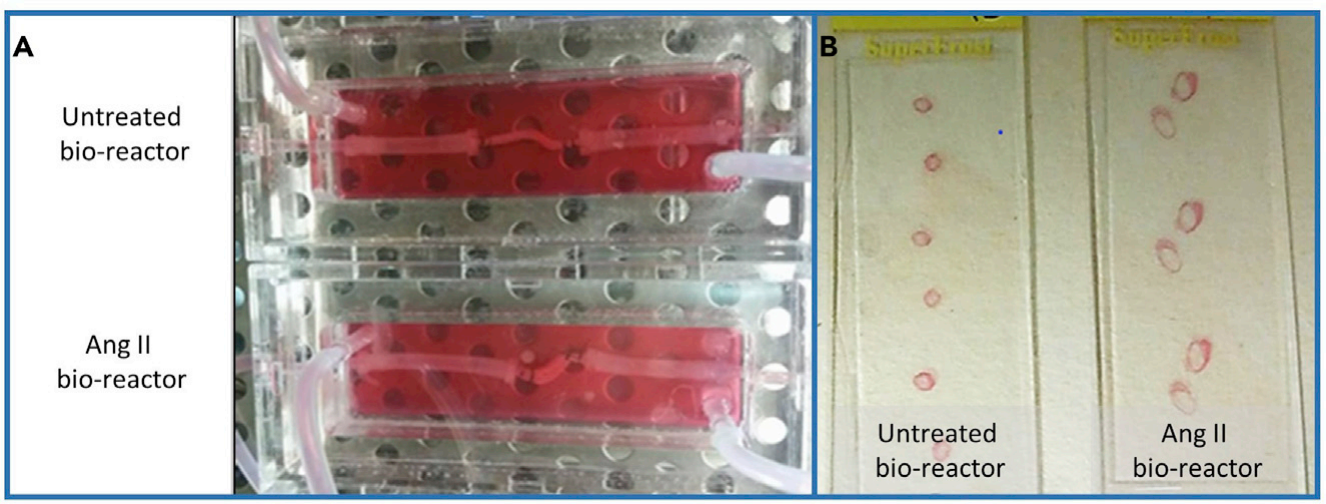
Representative Images of *Ex Vivo* Ang II-Infusion Aneurysm Model after 72 h within a Bioreactor Macroscopic changes in vessel dilatation were observed after 72 h within a bioreactor plus angiotensin II infusion compared to untreated control paired artery, assessed (A) while still within the bioreactor or (B) after histological sectioning and H&E staining.

As shown in Figure 4, umbilical cord arteries subjected to angiotensin II infusion for 72 h display marked macroscopically visible dilatation compared to a paired untreated control vessel. For the validation of our Ang II-infusion model, the umbilical cord artery was cut into three segments of equal length. One portion was flushed with PBS and inserted into a bioreactor system at 6.5 dyn/cm^2^ using media containing 5 μM Ang II in order to replicate the well-characterized mouse model of aneurysm formation. An equally sized length of the same umbilical cord artery was placed in the bioreactor system for 72 h at 6.5 dyn/cm^2^ with media alone and served as a paired control, termed untreated.

To ensure that the observed macroscopic changes were indicative of dilatation and therefore aneurysm formation, further histological examination was performed, as required to validate the model. As previously mentioned, the principle morphological and compositional characteristics associated with aneurysm formation were assessed, including vessel dilatation, medial thinning and associated elastin loss, and decreased medial vascular smooth muscle cell (VSMC) density.

**Figure 5:**
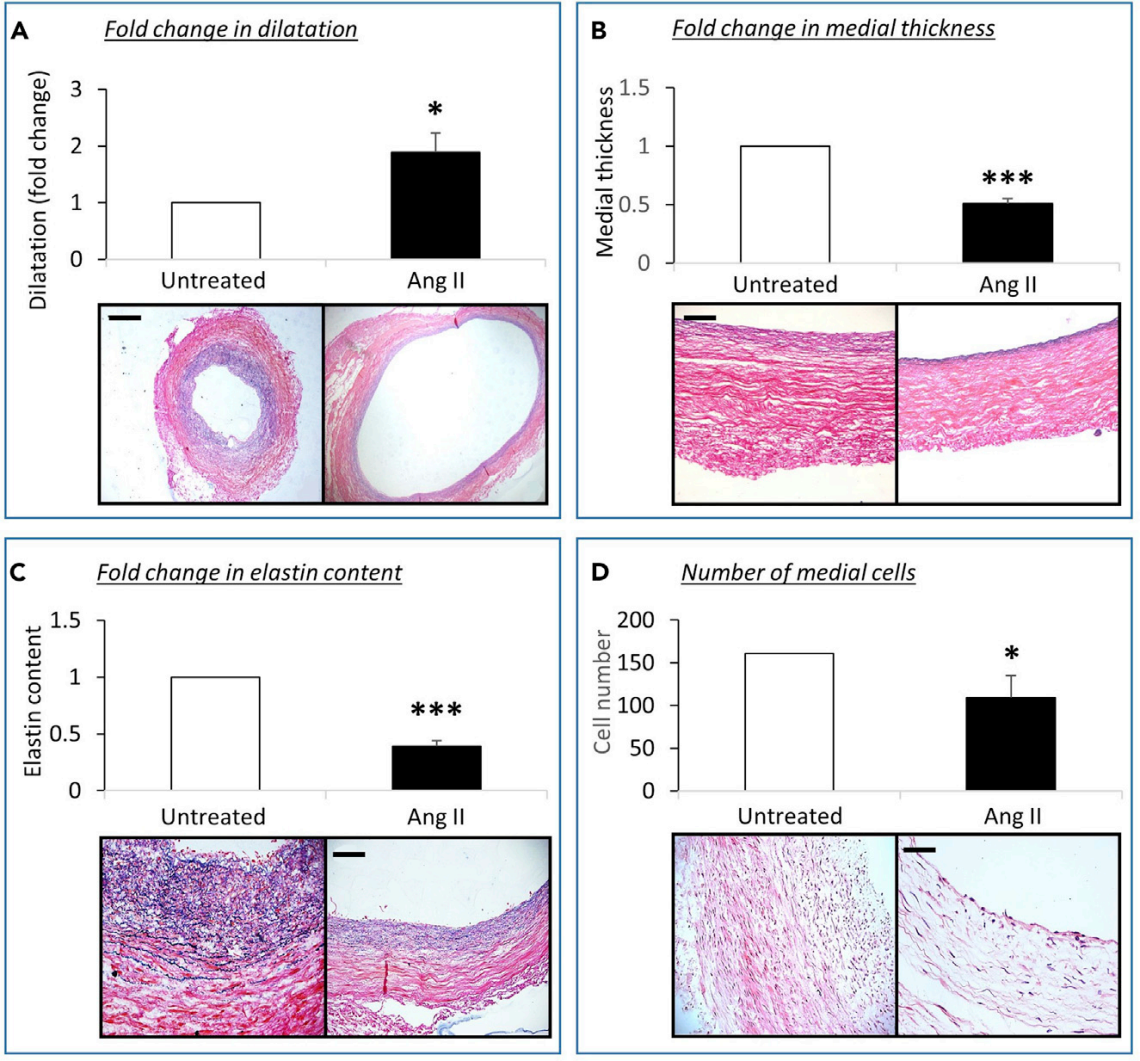
Effect of Ang II Administration on Arterial Vessel Dilatation, Medial Thickness, Elastin Content, and Medial Cell Number Quantification and representative images of (A) vessel dilatation, (B) medial thickness, (C) elastin content, and (D) number of medial cells assessed in ten ×20 magnification fields of EVG stained sections from human umbilical cord arteries after insertion within a bioreactor for 72 h without Ang II (untreated) and with Ang II (Ang II). Data are expressed as a fold change in Ang II-infused arteries compared to control (untreated) vessels (data are presented as mean ± SEM; n = 6). ∗∗∗p < 0.001, ∗p<0.05 versus control, 2-tailed Student paired t test. Scale bar represents 250 μm in (A) and 50 μm in (B)–(D).

As shown in Figure 5, Ang II infusion for 72 h under pulsatile laminar flow conditions demonstrated that the total vessel area (an indicator of dilatation) was significantly increased in Ang II-treated umbilical arteries (90%; P<0.05; n=6) compared to untreated paired control arteries. In addition, a significant reduction in umbilical artery medial thickness (49%; P<0.05; n=6), medial elastin content (60%; P<0.05; n=6), and medial cell density (32%; P<0.05; n=5) was observed in Ang II-treated umbilical arteries compared to paired untreated vessels. In summary, the above observations demonstrate our novel *ex vivo* bioreactor system employing arteries retrieved from human umbilical cord and subjected to Ang II administration, develops similar morphological and compositional changes associated with aneurysm formation as those observed in human aneurysms and mouse models. Assessment of these changes should be undertaken to validate the correct experimental setup of the model in new laboratory settings.

**Figure 6:**
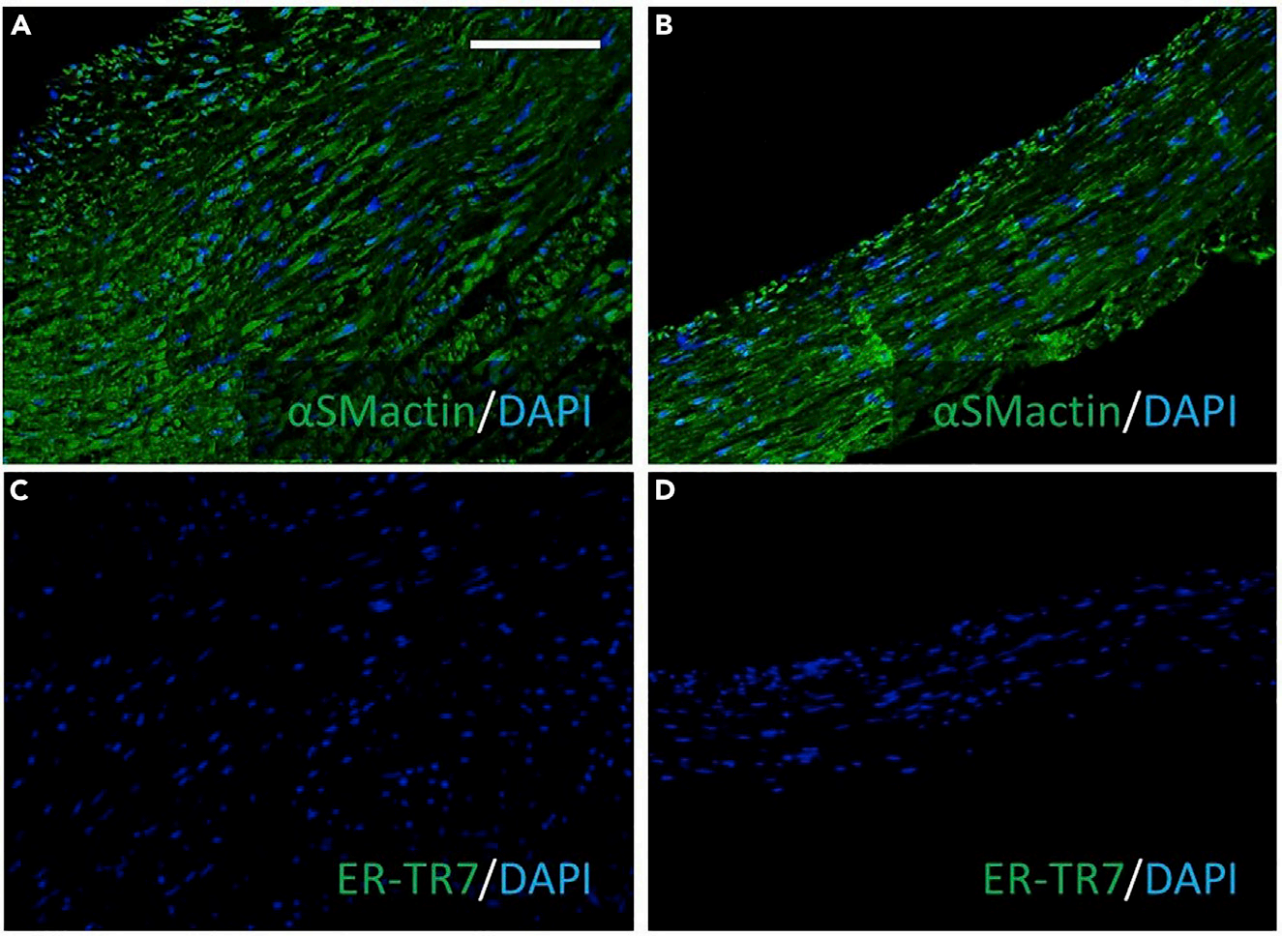
Effect of Ang II Administration on Arterial VSMC αSMactin and ER-TR7 Immunopositivity Representative images of immunohistochemistry for (A and B) alpha-smooth muscle actin (αSMactin; green color) alongside a DAPI nuclear label (blue), or (C and D) ER-TR7 (green color) alongside a DAPI nuclear label (blue), in sections from human umbilical cord arteries after insertion within a bioreactor for 72 h without Ang II (untreated; A and C) and with Ang II (Ang II; B and D). Scale bar in (A) represents 50 μm and is applicable to all panels.

Two additional related key indicators of aneurysm formation are VSMC phenotypic modulation and increased collagen turnover. VSMC modulation from a contractile phenotype to a synthetic form is characterized by a loss of contractile markers such as alpha-smooth muscle actin (αSMactin), which can be observed in umbilical cord arteries after Ang II administration within our ex vivo bioreactor model (Figure 6). However, very few fibroblasts (ER-TR7 positive) were detected (Figure 6).

**Figure 7:**
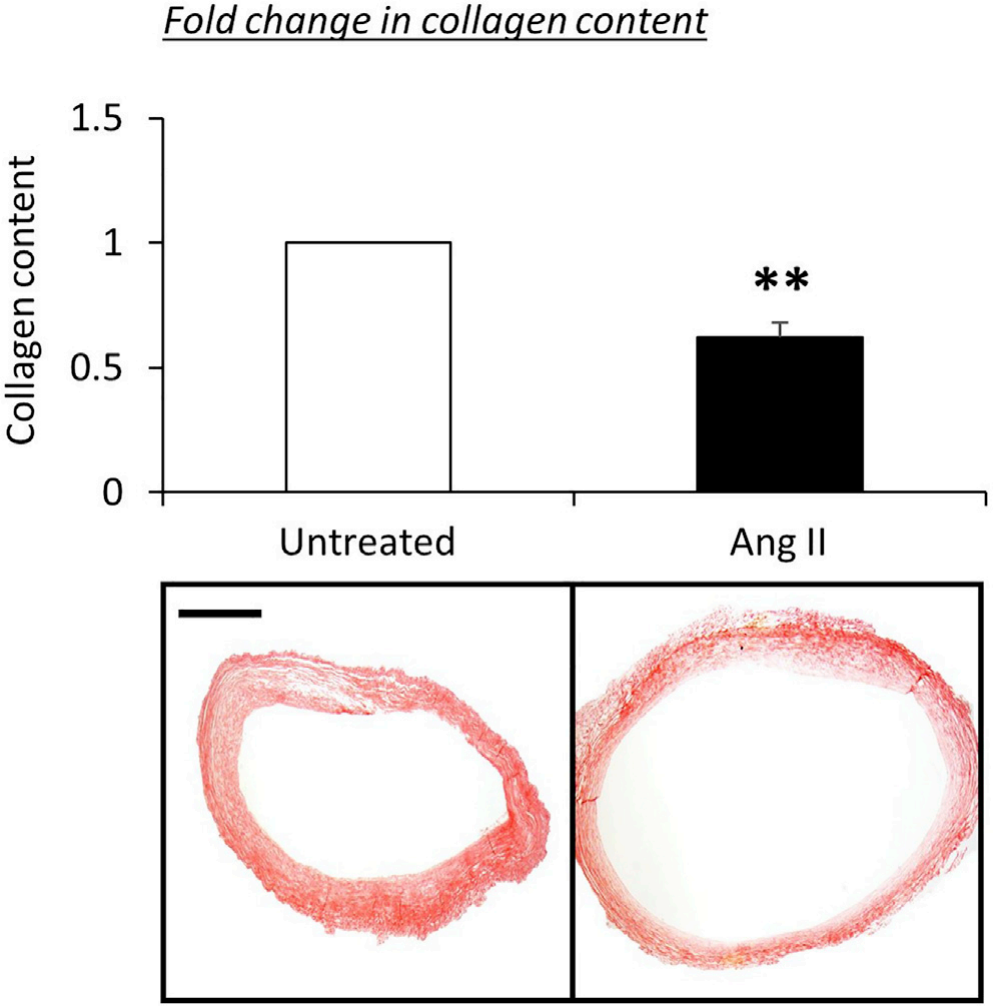
Ang II Administration Significantly Reduced Human Umbilical Cord Artery Fibrillar Collagen Content Compared to Untreated Control Arteries Quantification and representative images of total fibrillar collagen content assessed in ten ×20 magnification fields of Picrosirius red stained sections from human umbilical cord arteries after insertion within a bioreactor for 72 h without Ang II (untreated) and with Ang II (Ang II). Data are expressed as a fold change percentage in total collagen content of the Ang II-infused arteries compared to control (untreated) vessels (data are presented as mean ± SEM; n = 6). ∗∗p < 0.01 versus control, 2-tailed Student paired t test. Scale bar represents 100 μm and is applicable to both panels.

Increased collagen turnover is most commonly presented as a reduction in arterial wall total collagen content. Accordingly, analysis of picrosirius red stained sections and subsequent quantification demonstrates a reduction of total fibrillar collagen content (32%; P<0.05; n=5; Figure 7) is an additional robust marker and validative indicator of aneurysm formation within our ex vivo bioreactor model.

## Limitations

This model accurately reproduces key histological disease features observed in well-characterized animal models and the human pathology of aortic aneurysms, including vessel dilatation alongside compositional changes such as medial thinning, elastin degradation, and loss of VSMC content.

A potential limitation of the current model is the use of human umbilical cord arteries rather than the use of a human abdominal aorta. However, the structure and composition of the umbilical cord artery is similar to other arteries and it consists of three concentric layers: intima, media, and adventitia. The only marked difference is that umbilical arteries (similar to pulmonary arteries) carry deoxygenated blood which travels back to the placenta to be re-oxygenated again by the mother. The deoxygenated nature of the blood vessel (rather than oxygenated as is the case with the aorta) may affect the specific response to aneurysm inducers. However, the agreement in effects of Ang II on arterial changes in morphology and composition, between the *ex vivo* model and both human aneurysms and the currently available mouse models suggest a high degree of similarity. As such, human umbilical cord arteries represent a good alternative to human aortic tissue and are more readily available for use in aneurysm research experiments. Considering the majority of human abdominal aortic aneurysms display super-imposed atherosclerosis and marked inflammatory cell accumulation (Davies, 1998, Michel et al., 2011, Nordon et al., 2011), an additional limitation of the *ex vivo* model could be the absence of atherosclerosis-related inflammation. However, this is also a caveat with most mouse models of aneurysm and does not limit their use or publication in high impact journals. Moreover, the main intention of the *ex vivo* model is to replicate and ideally replace current animal models of aneurysm, while also providing a more translational platform through the use of human tissue.

## Troubleshooting

### Problem 1

The ethics used during the development of this model and its subsequent validation covered projects from three different research groups. As such, there are occasions when human umbilical cords are shared between research groups, which can limit both the size and volume of samples. For example, a specific number of umbilical cords permitted to be retrieved and used in research under the ethical permission can hamper large experiments.

### Potential Solution 1

If possible, have project-specific ethics in place which will allow use of the full length of the umbilical cord and ensure enough samples can be collected to fulfill the project requirements.

### Problem 2

The cross-section thickness of the umbilical cord artery can be different between donors as well as between arteries from the same umbilical cord.

### Potential Solution 2

We have to use a single umbilical cord artery and generate multiple segments from it at one time for control and intervention experiments. This will enable paired statistical analysis and reduce the intra-group variability resulting from the use of two different arteries of the same umbilical cord.

### Problem 3

During the setup of the model, the small silicon tube is connected with a cannula to the umbilical cord artery within a chamber system. The chamber system is then connected at two points to a long silicon tube that is attached to the peristaltic pump. These factors are important to close the system and permit the induction of laminar flow within an incubator. If the arterial wall is damaged or the artery is not tied appropriately to the cannula, there will not be any laminar flow.

### Potential Solution 3

In order to create consistent laminar flow, the arteries should be double tied at the two end points using appropriate surgical suture. If the arterial wall is damaged during this process, the damaged section should be discarded, and a new section of the same artery should be used.

## Resource Availability

### Lead Contact

Further information and requests for resources and reagents should be directed to and will be fulfilled by the Lead Contact, Jason Johnson (jason.l.johnson@bristol.ac.uk).

### Materials Availability

This study did not generate new unique reagents.

### Data and Code Availability

This study did not generate any unique datasets or code.
